# A large cystic meningioma incidentally detected during general examination for breast cancer

**DOI:** 10.1016/j.radcr.2022.03.001

**Published:** 2022-03-26

**Authors:** Hiroki Sugiyama, Satoshi Tsutsumi, Aito Watanabe, Senshu Nonaka, Hidehiro Okura, Hiroshi Izumi, Hisato Ishii

**Affiliations:** a Department of Neurological Surgery, Juntendo University Urayasu Hospital, Urayasu, Chiba, Japan; b Department of Pathology, Juntendo University Urayasu Hospital, Urayasu, Chiba, Japan

**Keywords:** Cystic tumor, Cystic meningioma, Breast cancer, Treatment

## Abstract

A 57-year-old woman who underwent needle biopsy for a subcutaneous mass in the breast was diagnosed with invasive ductal carcinoma. General examination incidentally revealed an intracranial tumor. At presentation, the patient showed memory disturbance but no focal neurological deficits. Cranial computed tomography (CT) revealed a large, hypodense cyst in the left frontotemporal region, involving a tumor with extensive hyperostotic changes in the left sphenoid and frontal bones. Magnetic resonance imaging showed that the tumor was attached to the dura mater of the pterional region and extensively enhanced, with involvement of the frontal and sphenoid bones. The less vascular tumor was removed en bloc by drilling the affected sphenoid and frontal bones as much as possible. The microscopic findings of the tumor were consistent with meningothelial meningioma with invasion into the dura mater and bone. Cystic meningioma should be considered when encountered with a dural-based cystic tumor, even in patients with cancer. In such circumstances, prompt and preferential resection may be indicated for intracranial tumors for timely initiation of the long-term treatment of cancer.

## Introduction

Meningiomas are the most common type of primary non-glial brain tumors. Infrequently, meningiomas accompany intra- or perilesional cysts and are called cystic meningiomas (CMs) [1], [2], [3]. Radiologically, CMs may be difficult to differentiate from malignant brain tumors or other intracranial cysts, particularly when they are intraparenchymally located. CMs are usually benign, low-grade tumors [3], [4], [5]. However, enhancement of the cyst walls highly suggests of inclusion of tumor cells. Therefore, in such circumstance, cyst resection is recommended [2,3,6,7].

The lesser sphenoid wing is the third most frequent original sites of intracranial meningiomas. Meningiomas arising from it have characteristics of both skull base and convexity meningiomas. Their management is challenging owing to their frequent and extensive bone invasion, in addition to marked hyperostosis and proximity to essential neurovascular structures [8], [9], [10], [11], [12]. While these meningiomas are thought to be commonly low-grade tumors, a recent study reported that they tended to be higher-grade, harboring neurofibromatosis type 2 mutations [8,13].

Here, we report a case of CM arising from the lateral part of the lesser sphenoid wing with extensive hyperostotic changes incidentally detected during a general examination for breast cancer yet to be treated.

## Case report

**Fig. 1:**
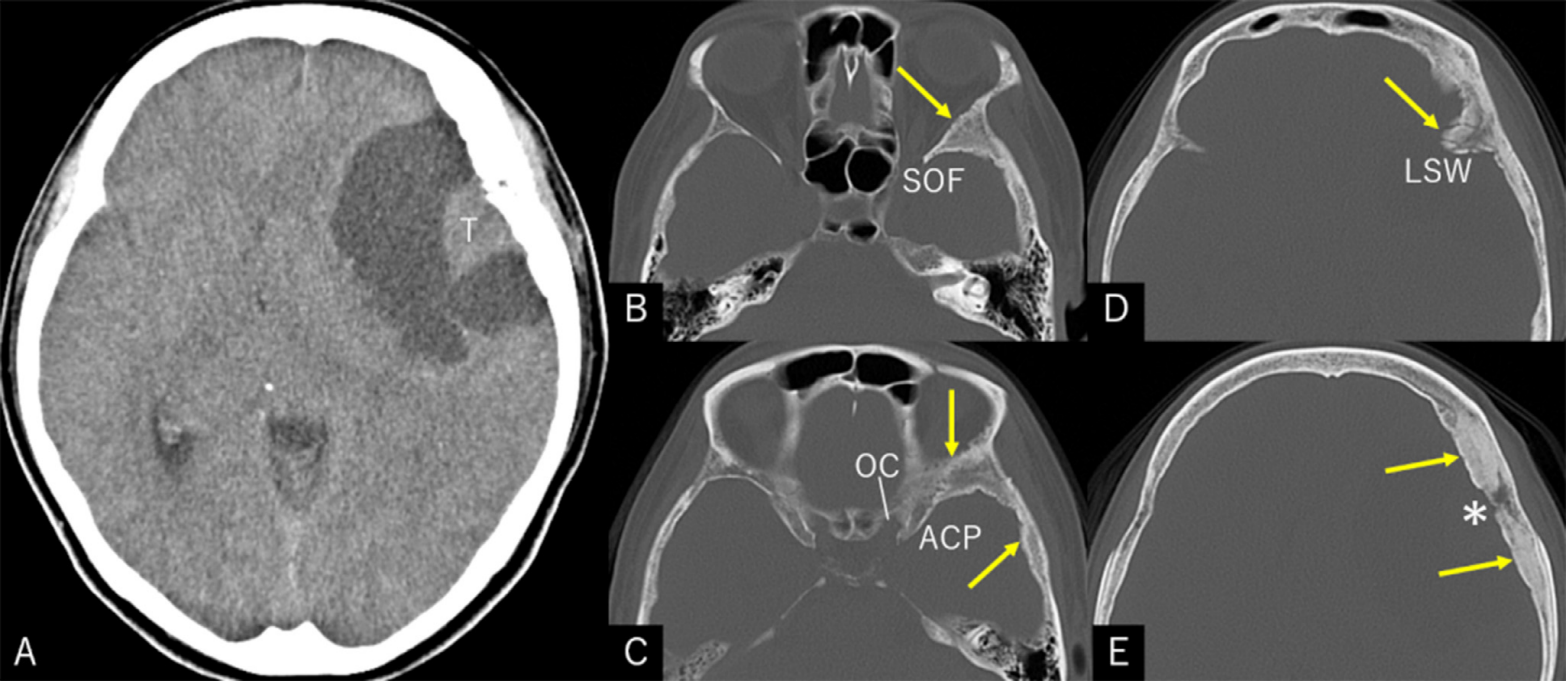
(A) Non-contrast axial computed tomography (CT) scan showing a hypodense cyst in the left frontotemporal region with an isodense tumor included in it. (B–E) Axial bone-target CT scans at the level of the superior orbital fissure (B), anterior clinoid process (C), upper part of the lesser sphenoid wing (D), and the pterion (E) showing extensive hyperostotic changes involving the left sphenoid and frontal bones (*arrows*). The morphologies of the superior orbital fissure and optic canal are intact. *ACP:* anterior clinoid process; *LSW:* lateral part of the lesser sphenoid wing; *OC:* optic canal; *SOF:* superior orbital fissure; *T:* tumor; *Asterisk:* site of tumor attachment.

**Fig 2:**
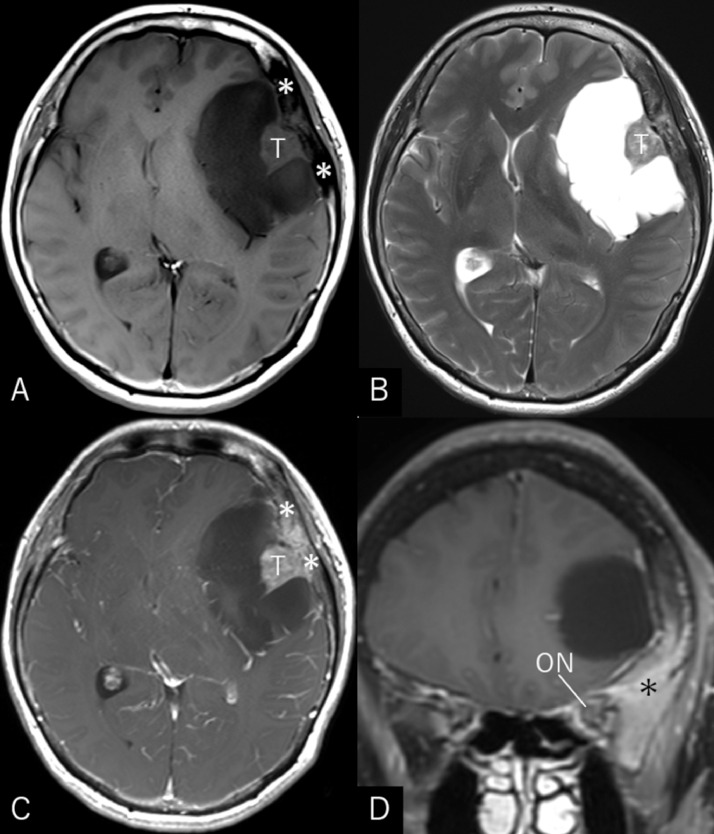
Axial T1- (A), T2-weighted (B) magnetic resonance (MR) images showing a well-demarcated, apparently extra-axial cyst 68 × 46 × 48 mm in maximal dimensions and involving a tumor (*T*) 16 × 20 × 24 mm in maximal dimensions. The tumor shows accompanying cyst components and is attached to the dura mater of the pterional region. Post-contrast axial (C) and coronal (D) MR images showing inhomogeneous enhancement of the tumor and adjacent diploe with extensive involvement of the frontal and sphenoid bones (*asterisk*), while the wall of the cyst is not enhanced. *ON:* optic nerve.

**Fig 3:**
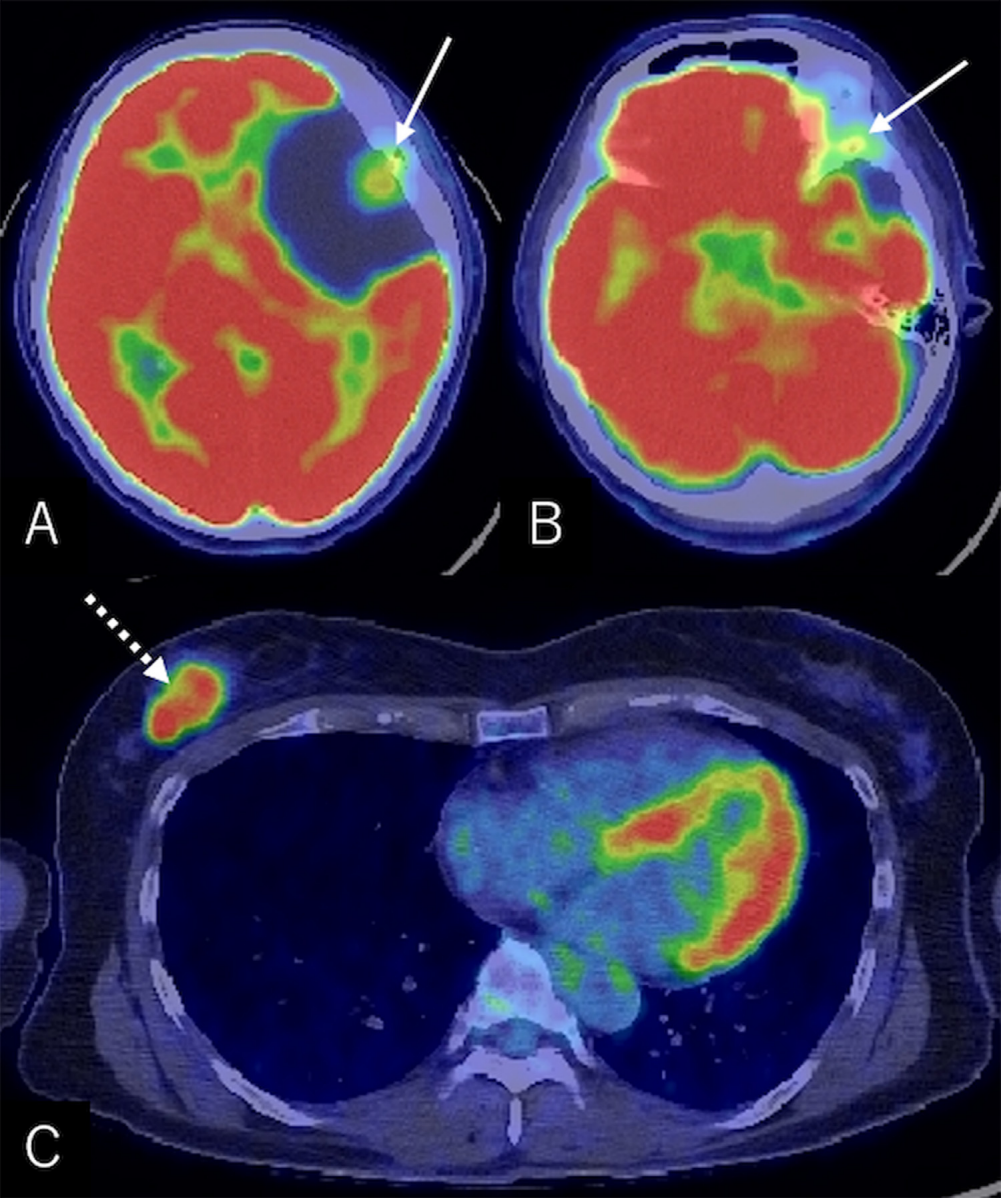
(A–C) Axial F-18 fluorodeoxyglucose positron emission tomography/computed tomography scans showing abnormal, but less considerable accumulations in the intracranial tumor and adjacent frontal and sphenoid bones (A and B*,* arrow) compared to the right breast cancer (C*,* dashed arrow).

**Fig 4:**
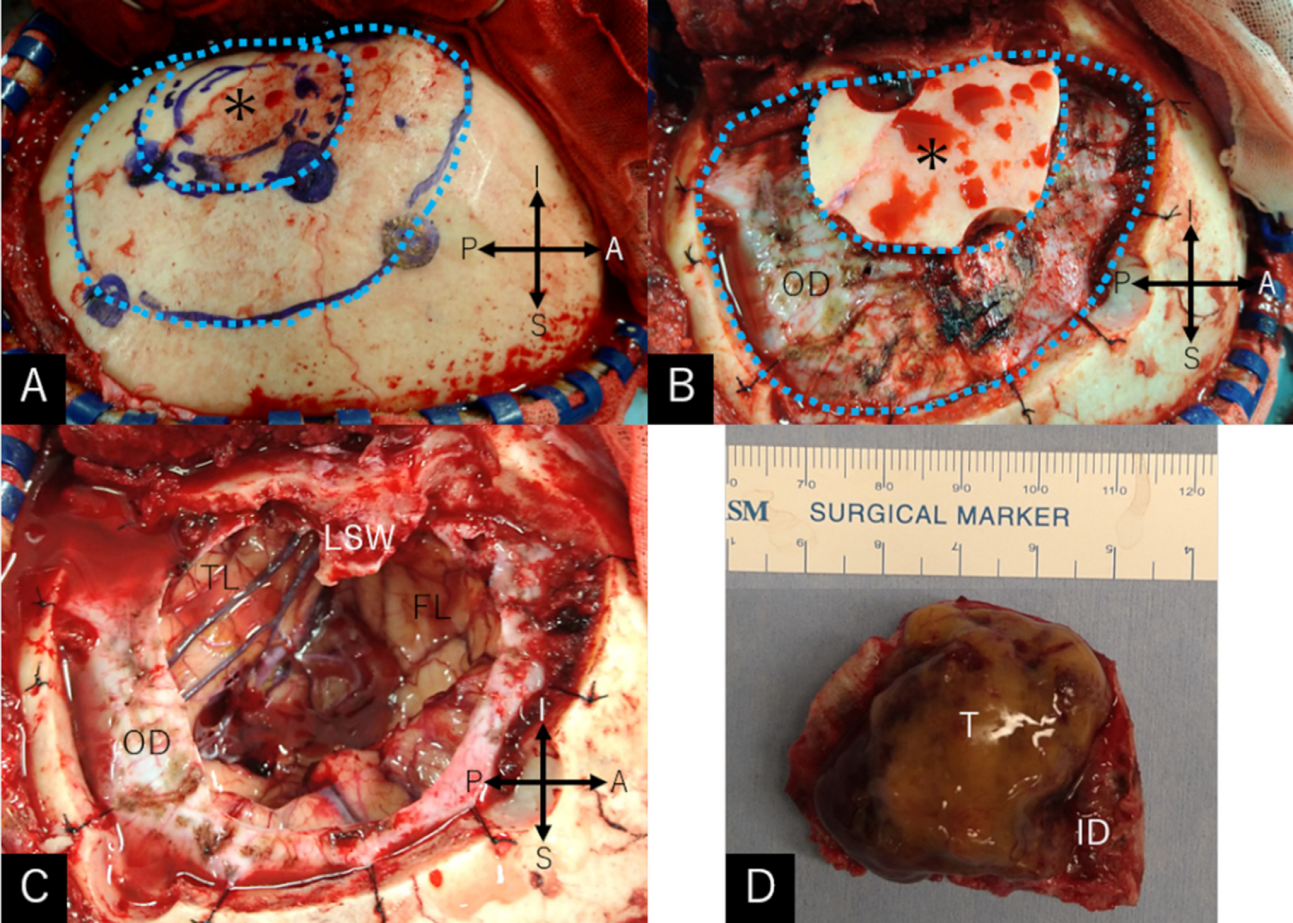
Intraoperative photos showing the ranges of double craniotomies comprised by inner and outer bone flaps (A), appearance after cutting off the outer bone flap (B), appearance after tumor resection with the affected dura mater and inner bone flap (C), and inner surface of the tumor with attached dura mater (D). *A:* anterior; *FL:* frontal lobe; *I:* inferior; *ID:* inner surface of the dura mater; *LSW:* lateral part of the lesser sphenoid wing; *OD:* outer surface of the dura mater; *P:* posterior; *S:* superior; *T:* tumor; *TL:* temporal lobe; *Asterisk:* inner bone flap with tumor attachment underneath; *Dashed lines:* craniotomy lines of the outer bone flap.

**Fig 5:**
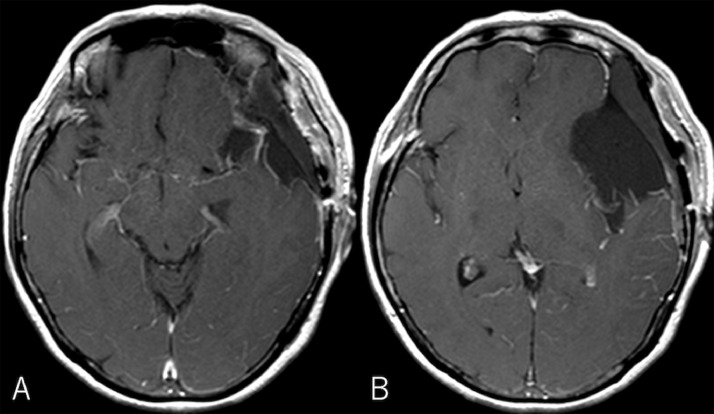
Post-contrast axial magnetic resonance images showing a comfortable resection of the tumor.

A 57-year-old, right-handed woman was aware of a subcutaneous mass in her right breast that had persisted for 6 months. She underwent needle biopsy and was diagnosed with invasive ductal carcinoma. The general examination incidentally revealed a large intracranial tumor, and the patient was referred to our department. At presentation, she did not show any focal neurological deficits or headache; however, a recent memory disturbance was detected by Hasegawa dementia scale-revised (HDS-R), with a total score of 21/30. Cranial computed tomography (CT) revealed a hypodense cyst in the left frontotemporal region with an isodense tumor (Fig. 1A). The bone-target images showed extensive hyperostotic changes involving the left sphenoid and frontal bones. The morphologies of the superior orbital fissure and optic canal were intact (Fig. 1B-E). Cerebral magnetic resonance imaging (MRI) showed a well-demarcated, apparently extra-axial cyst measuring 68 × 46 × 48 mm in maximal dimensions and involving a tumor 16 × 20 × 24 mm in size. The tumor was accompanied by cyst components and attached to the dura mater of the pterional region. Post-contrast images showed extensive enhancement of the tumor and adjacent diploe, involving the frontal and sphenoid bones, while the cyst wall was not enhanced (Fig. 2). No other intracranial lesions were noted. F-18 fluorodeoxyglucose positron emission tomography (PET)/CT scans showed abnormal but less intense accumulations in the intracranial tumor and adjacent frontal and sphenoid bones, compared to the right breast cancer (Fig. 3). With a presumptive diagnosis of CM, the patient underwent tumor resection through double craniotomies, comprising inner and outer bone flaps in the left frontotemporal region (Fig. 4A). After cutting off the outer bone flap (Fig. 4B), the less vascular tumor was removed en bloc with the attached dura mater and inner bone flap by making circumferential cuts (Fig. 4C, D). The hyperostotic frontal bone and apparently affected, fragile cancerous bones of the lesser sphenoid wing were drilled as much as possible. The cyst walls, comprising arachnoid-like transparent membranes, were left unresected. Post-operative MRI confirmed comfortable resection of the tumor (Fig. 5).

**Fig. 6:**
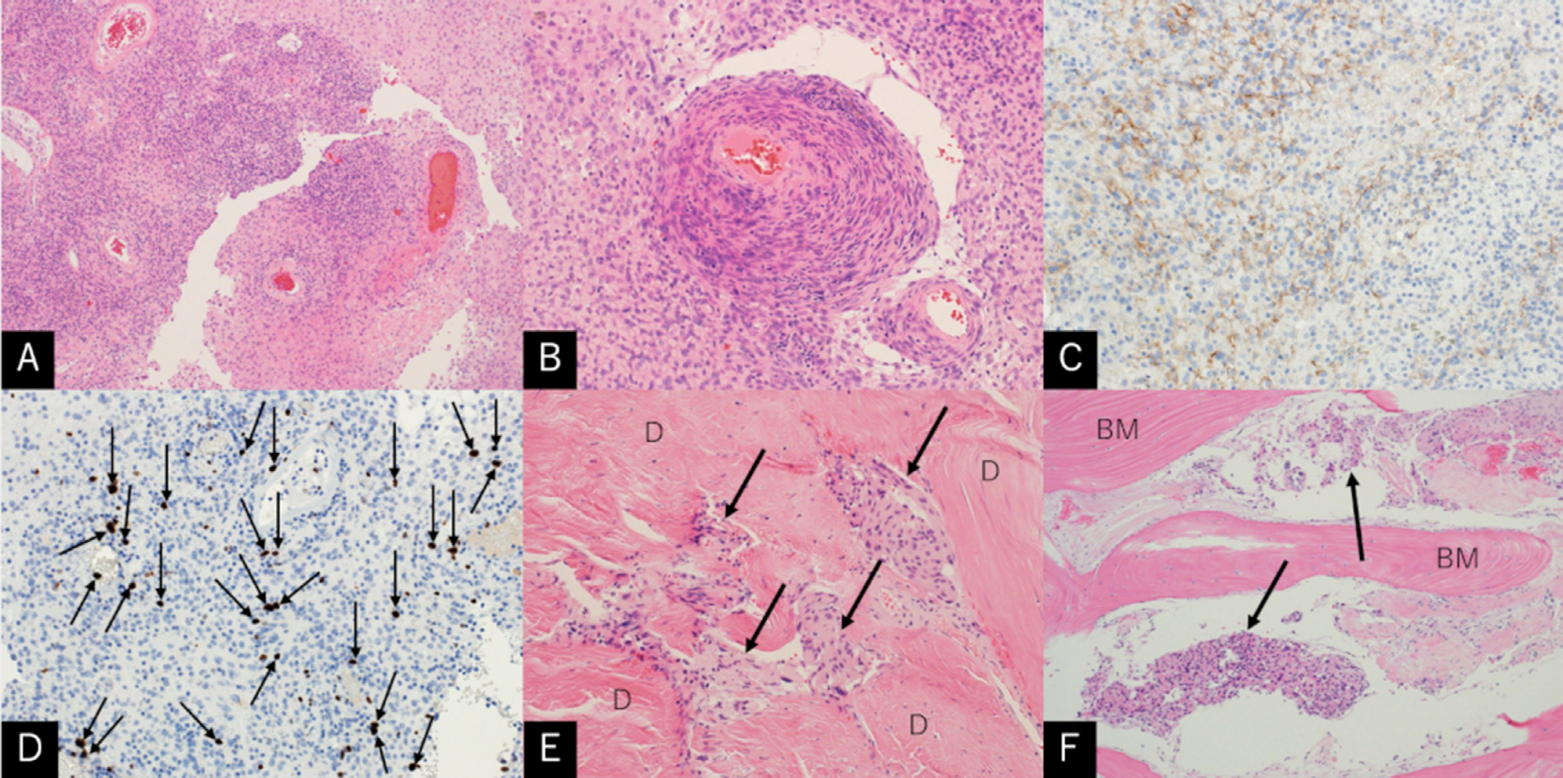
Photomicrographs of the resected specimens showing sheet-like proliferation of arachnoid-cell like, neoplastic cells with angiogenesis (A, B). The cells are negatively stained for epithelial membrane antigen (C) with a MIB-1 index of 5% (D). Invasions into the dura mater (E) and bone (F) in the superolateral part of the lesser sphenoid wing are also visible. BM: bone matrix; D: dura mater; A, B, E. F: hematoxylin and eosin stain, A: x40; B: x200; E: x200; F: x100; C: epithelial membrane antigen stain, x200; D: Ki-67 stain, x200.

Microscopically, tumor tissue comprised by sheet-like proliferation of arachnoid cell-like, neoplastic cells accompanied by angiogenesis. Staining for epithelial membrane antigen was negative and the MIB-1 index was 5%. Furthermore, invasion of the adjacent dura mater and bone was observed (Fig. 6). There were no findings of metastatic breast cancer. The patient's postoperative course was uneventful. Her HDS-R score performed on postoperative day 4 was 26 of 30 with improvement in memory disturbance. The patient is currently planning to undergo resection of the breast cancer.

## Discussion

CMs are occasionally difficult to differentiate from malignant brain tumors and other intracranial cysts [3], [4], [5]. In the present case, a large cystic tumor was incidentally detected during the general examination for breast cancer. Based on its large size for patient's subtle neurological impairments, dural-based appearance, accompaniment of intra- and perilesional cysts, and extensive hyperostotic changes on CT and MRI, and less intense accumulation compared to breast cancer on PET/CT scans, we considered the tumor could be a low-grade CM, rather than a metastatic tumor, as the most probable presurgical diagnosis.

Regarding its original site, the tumor shared the characteristics of sphenoid wing meningiomas [8], [9], [10], [11], [12]. Dalle et al. reported in a large series that a total resection of sphenoid wing meningiomas with extensive hyperostosis was carried out only in 43% of 54 patients, while those meningiomas were histologically World Health Organization grades I and II in 85% and 15%, respectively. In their series, postoperative complications occurred in 44% of patients, and tumor recurrence or progression was found in 22% [9]. In our case, symptoms caused by a large CM were subtle for the patient. Despite extensive hyperostotic changes in the lesser sphenoid, morphologies of the superior orbital fissure and optic canal were intact. Commonly, these meningioma-associated bony changes are caused by low-grade tumors [8]. Furthermore, the breast cancer in the patient was yet to be treated. Therefore, we performed a less aggressive surgery for the CM to facilitate the prompt initiation of breast cancer treatment.

Although the present CM was thought to be an incidental coexistence with a breast cancer, metastasis of breast cancer to intracranial meningiomas and their simultaneous presentation have been reported [14,15]. Lieu et al. proposed that intracranial lesions in patients with breast cancer should not be immediately labeled as metastases and that meningiomas should be excluded [15].

In conclusion, CM should be considered when encountered with dural-based cystic tumors, even in patients with cancer. In such circumstances, prompt and preferential resection may be indicated for intracranial tumors to allow timely initiation of the long-term treatment of cancer.

## Author Contributions

All the authors contributed equally to this study.
